# Retraction Note: Cardamonin induces ROS-mediated G2/M phase arrest and apoptosis through inhibition of NF-κB pathway in nasopharyngeal carcinoma

**DOI:** 10.1038/s41419-019-1482-8

**Published:** 2019-03-27

**Authors:** Yuting Li, You Qin, Chensu Yang, Haibo Zhang, Yong Li, Bian Wu, Jing Huang, Xiaoshu Zhou, Bo Huang, Kunyu Yang, Gang Wu

**Affiliations:** 1 Cancer Center, Union Hospital, Tongji Medical College, Huazhong University of Science and Technology, Wuhan, 430022 China; 2 Department of Radiotherapy, Zhejiang Province People’s Hospital, Hangzhou, Zhejiang China; 3 Department of Oncology, Renmin Hospital, Hubei University of Medicine, Shiyan, Hubei China; 4 Department of Biochemistry and Molecular Biology, Tongji Medical College, Huazhong University of Science and Technology, Wuhan, Hubei China; 5 State Key Laboratory of Medical Molecular Biology and Department of Immunology, Institute of Basic Medical Sciences, Chinese Academy of Medical Sciences and Peking Union Medical College, Beijing, China

**Retraction to: Cell Death & Disease** 8:e3024

10.1038/cddis.2017.407

published online: 31 August 2017

The authors have retracted this article because of numerous errors in the figures. Specifically:

1. In Figure 1a, panel CNE-1, HONE-1 and SUNE-1 are duplicated.

2. In Figure 1b, CNE-1, CNE-2 and HONE-1 are duplicated.

3. In Figures 2c, 2e, 3i, and 4b, the β-actin loading controls are identical.

4. In Figures 2e (left) and 4b, the β-actin loading controls are identical.

5. In Figures 3f and 3h, the β-actin loading controls are identical.

6. In Figure 3i, the western blot molecular mass labels are incorrect.

7. Figure 4c is incorrectly labelled as NF-κB immunofluorescence.

8. The scales of measurement for Figures 5a and 5b are incorrect.

All authors agree to this retraction.

